# Understanding the marginal distributions and correlations of link travel speeds in road networks

**DOI:** 10.1038/s41598-020-68810-9

**Published:** 2020-07-16

**Authors:** Feng Guo, Xin Gu, Zhaoxia Guo, Yucheng Dong, Stein W. Wallace

**Affiliations:** 1 Business School, Sichuan University, Chengdu, 610065 China; 2 NHH Norwegian School of Economics, Bergen, Norway

**Keywords:** Statistical physics, Information theory and computation

## Abstract

Link travel speeds in road networks are essential data for a variety of research problems in logistics, transportation, and traffic management. Real-world link travel speeds are stochastic, and highly dependent on speeds in previous time periods and neighboring road links. To understand how link travel speeds vary over space and time, we uncover their distributions, their space- and/or time-dependent correlations, as well as partial correlations, based on link travel speed datasets from an urban road network and a freeway network. We find that more than 90% (57%) of travel speeds are normally distributed in the urban road (freeway) network, and that correlations generally decrease with increased distance in time and space. We also investigate if and how different types of road links affect marginal distributions and correlations. The results show that different road link types produce quite similar marginal distributions and correlations. Finally, we study marginal distributions and correlations in a freeway network. Except that the marginal distribution and time correlation are different from the urban road network, others are similar.

## Introduction

With advances in information technology, the volume of data is growing at an unprecedentedly rapid rate. The power of big data has been observed in various real-world applications^1-3^. In the traffic and transport sector, vast amounts of data makes it possible to reveal certain facts about real-world traffic and transport operations. Some examples reported involve understanding traffic capacity^4^, congested travel^5^ and road usage patterns^6^ in urban road networks.

Link travel speeds in road networks are fundamental for a variety of decision-making problems in logistics, transportation and traffic^7^. Link travel speeds change over space and time due to such as changing travel demands and unstable traffic flows. There is an increased use of stochastic travel speeds (times) in various real-world decision-making problems such as vehicle routing^8-12^, shortest or reliable paths^13-17^, ridesharing^18,19^, traffic assignment^20,21^ and traffic or travel time (speed) predictions^22-24^. As two important indicators to describe stochastic travel speeds, marginal distributions^8-13,15-17,21,23^ and correlations^9,13-17,22-24^ have been widely used in these studies.

Considering correlated stochastic travel speeds in shortest path problems could lead to a carbon emission reduction from 0.02 to 0.20%^13^. For reliable path problems, the inaccuracy in the reliability objective is 15% on average without the consideration of correlations in link travel time^16^. However, these studies usually assume that the stochastic travel speeds obey certain continuous^10-12,15-17,21^ or discrete^9,14,24^ distributions and have some simple^9,14-16^ or even no correlations^10,21^.

Some researchers have investigated marginal distributions^25,26^ or correlations^23,27-29^ of travel speeds (times) on the basis of chosen road links and a limited number of travel speed data. By analyzing a 14-km tollway in Melbourne, Australia, it is found that the distribution of travel time changes from log-normal to normal when the number of counted vehicles decreases^25^. The data collected at a location on I-35 in Austin shows that travel times tend to be normally distributed in moderate traffic conditions but lognormal under congestion^26^. The increase in distance tends to reduce the correlation of travel speeds (times)^23,27,29^, while higher congestion usually leads to higher correlations^27^. By studying 40 links in the urban area of ​​Philadelphia, it is shown that the network structure plays a large role in spatiotemporal correlations in link travel speeds in a network^28^. However, these findings could be misleading since they are only based on a limited number of road links and insufficient data.

By examining the correlations of immediately neighboring links in space and time based on a 45-day travel speed dataset, significant spatial correlations were observed for about 50% of the links and their adjacent links in the same direction, while travel speeds between two consecutive time periods exhibited strong significant temporal correlations^7^. By investigating temporal correlations and spatial correlations of 30 randomly chosen links from the California freeway network based on a 102-day travel speed dataset^17^, it is found that, the temporal correlation of travel speeds on one link decreases on the whole with the increase of time period lag. However, it is unclear if the same findings can be made if more links are considered.

For real-world link travel speeds, the correlation between any two random speed variables could provide misleading results because speeds on other links could be numerically related to both variables. To avoid this misleading information, we can calculate partial correlations by removing the effect of a set of controlling random variables. However, various partial correlations in space and/or time have not been reported yet. It is thus worthwhile to investigate and understand real marginal distributions and correlations of link travel speeds from the perspective of a large-scale road network.

**Figure 1:**
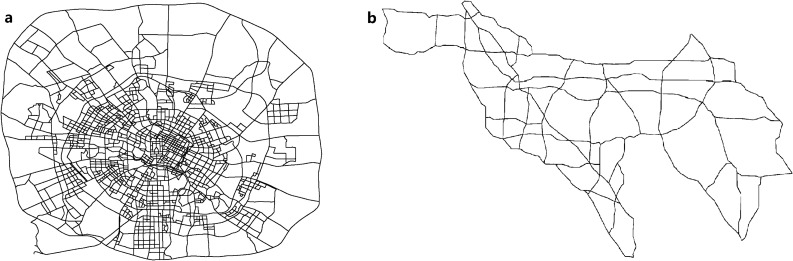
Two road networks used. (**a**) Urban road network of Chengdu (Figure courtesy of Guo et al.^7^). (**b**) Freeway network of California.

To fill this gap, we, for the first time, address the marginal distributions and various correlations of travel speeds in a large-scale urban road network (Fig. 1a) and a freeway network (Fig. 1b). The urban road network is from a megacity in Western China, Chengdu, which contains 1,902 nodes and 5,943 directed links. The dataset from this network involves 1,782,900 random variables (link travel speeds) and 5.5 billion Global Positioning System (GPS)-enabled raw trajectory data. The freeway network contains 168 nodes and 438 directed links, the dataset for which involves 31,536 random variables and 51 million raw travel speeds from 3,417 speed detection stations of the California freeway network. We consider temporal, spatial, and spatial-temporal correlations and their partial versions.

## Results

### Marginal distributions of link travel speeds

**Figure 2:**
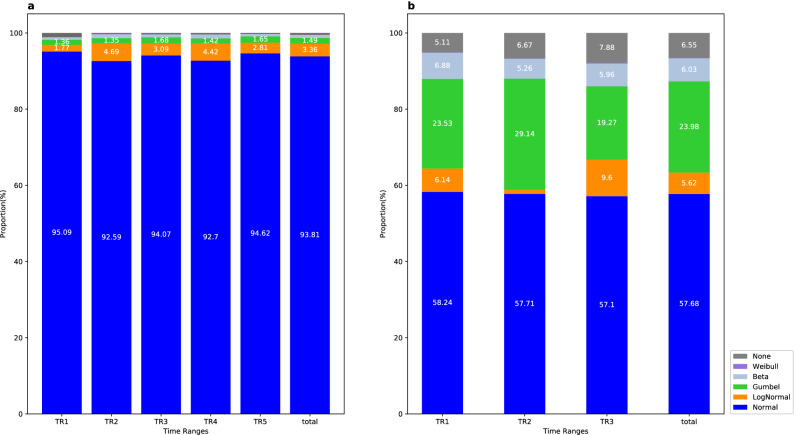
Proportions of different distribution types in different time ranges. Different colors in each bar represent the proportions of different distribution types. The number in each color indicates the percent corresponding to this color. Five best-fitting distributions, that can fit more than 0.1% of travel speed variables in either road network type, are identified. ‘None’ indicates the proportions of travel speeds that do not follow any of the 4 distributions. (**a**) Urban road network. (**b**) Freeway network.

As Figure 2 shows, in the urban road network, 93.81% (1,672,538 random variables) of link travel speeds are normally distributed, while the lognormal, the Gumbel, the beta, and the Weibull distributions cover 3.36%, 0.11%, 1.49% and 0.81% respectively. Only 0.52% of random speed variables fit none of these distributions. In the freeway network, the normal, the lognormal, the Gumbel, the beta, and the Weibull distributions cover 57.68%, 5.62%, 23.98%, 6.04%, and 0.13%, respectively. The remaining 6.55% do not follow any of these distributions. For the freeway network, much fewer link travel speeds are normally distributed. The marginal distributions are relatively similar in different time ranges (See Supplemental Table S1). The percentage of normal distributions in different time periods ranges between 0.11 and 2.5% in two networks. For lognormal, Gumbel, beta, and Weibull distributions, the percentages range within [0.27%, 2.92%], [0.01%, 0.33%], [0.03%, 0.43%], and [0, 0.01%] respectively in the urban road network, while they range within [3.46%, 8.48%], [4.26%, 9.87%], [0.7%, 1.62%], and [0, 0.09%] respectively in the freeway network.

### Temporal correlations of link travel speeds

**Figure 3:**
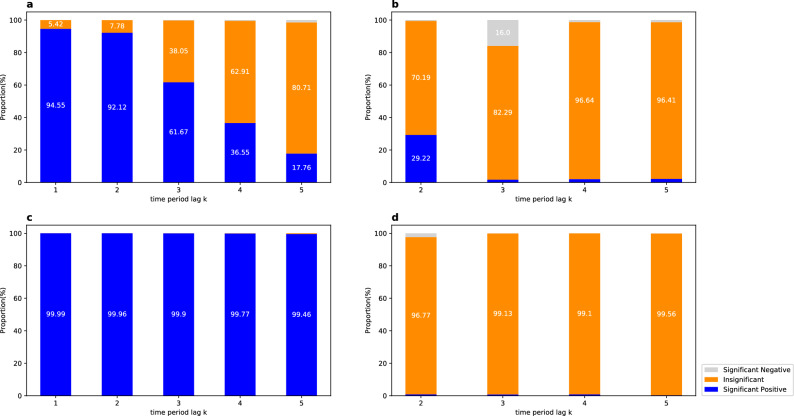
Proportions of temporal correlations and temporal partial (TP) correlations of travel speeds between period $i$ and period $i + k\left( {k = 1,2, \ldots 5} \right)$. The three colors in each bar represent the average proportions from different time ranges at a specific time period lag, for significant positive, insignificant, and significant negative temporal or TP correlations, respectively. We provide the average results of different time ranges here because different time ranges have little effect on the results of temporal and TP correlations (see Supplemental Tables S2 and S3). (**a**) Urban road network (temporal). (**b**) Urban road network (TP)**.** (**c**) Freeway network (temporal). (**d**) Freeway network (TP).

As shown in Fig. 3, for the urban road network, more than about 90% of travel speeds are significantly positively correlated with a time period lag of $k \le 2$. When $k$ increases from 2 to 5, the number of significant positive correlations decreases substantially from 92.12 to 17.76%, whereas the number of insignificant correlations increases from 7.78 to 80.71%. For all time ranges and time period lags, significant negative correlations are rare. Specifically, less than 1.54% of correlations are significantly negative at $k \le {4}$. More significant positive temporal correlations (> 99.46% in total) are observed in the freeway network, because the traffic flow is smoother and the speed fluctuations over time are smaller. Generally speaking, the correlations are very close in different time ranges (See Supplemental Table S2). Taking $k = 1$ as an example, the percentages of significant positive (negative) correlations range within [91.78%, 97.75%] ([0.02%, 0.1%]) in the urban road network, while more than 99.99% of the temporal correlations are significantly positive in the freeway network. In addition, the morning and evening rush hours generate more significant positive correlations than non-rush hours do at $k > 3$.

### Temporal partial (TP) correlations of link travel speeds

As Fig. 3 shows, for the urban road network, 29.22% of TP correlations are significantly positive between period $i$ and period $i{ + }2$ whereas only less than 2.19% can be observed at $k > 2$; and more than 96% of TP correlations are insignificant at $k > 3$. For the freeway network, more than 96.77% of TP correlations are insignificant at $k \ge 2$, while significant positive and significant negative correlations are almost negligible. When the time lag is sufficiently large (say 8 or 10 min), almost all TP correlations are insignificant. It is worth noting that about 16.0% of TP correlations are significantly negative at $k = 3$, which is much larger than the ones for other $k$ and probably caused by traffic signal lights.

### Spatial correlations of link travel speeds

**Figure 4:**
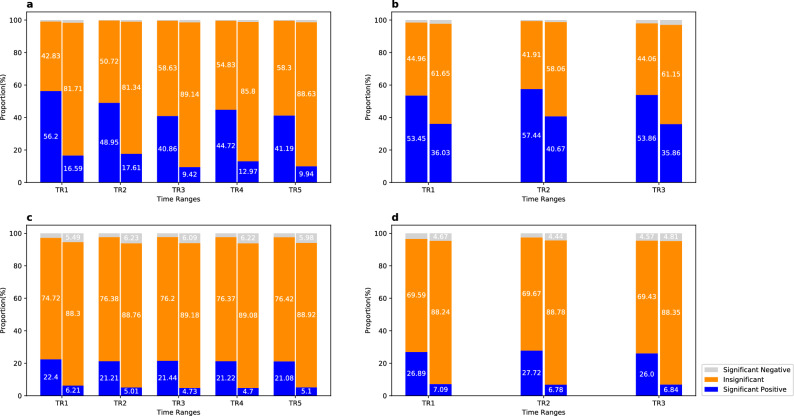
Proportions of spatial correlations and spatial partial (SP) correlations of travel speeds in different time ranges for both directly and indirectly adjacent cases. The three colors in each bar represent the average proportions from different time periods within a specific time range, for significant positive, insignificant, and significant negative spatial or SP correlations, respectively. The two bars for each time range represent the directly adjacent (left) and indirect adjacent cases (right) respectively. We provide the average results of different time periods here because different time periods have little effect on spatial and SP correlation (see Supplemental Tables S4 and S5). (**a**) Urban road network (spatial). (**b**) Freeway network (spatial). (**c**) Urban road network (SP). (**d**) Freeway network (SP).

In each 2-hour time range, we calculate the spatial correlations of travel speeds on all links in 6 different time periods for the urban road network (i.e., periods 5, 15, 25, 35, 45 and 55) and the freeway network (i.e., periods 2, 6, 10, 14, 18 and 22). Figure 4 shows the results from all these periods. Consider the urban road network first. Significant negative spatial correlations for directly and indirectly adjacent cases occupy less than 0.97% and 1.70%, respectively. Two links are directly adjacent if they share a node and have the same traffic-flow direction. They are indirectly adjacent if the two links share a node but have different traffic-flow directions, or the two links have the same traffic-flow direction and connect to different nodes of the same link. The number of significant positive correlations and the number of insignificant correlations are comparable for directly adjacent cases, whereas the majority (81.34–89.14%) of correlations are insignificant for indirectly adjacent case. For the freeway network, significant negative spatial correlations for directly and indirectly adjacent cases account for less than 2.08% and 2.99%, respectively. The numbers of insignificant (significant positive) spatial correlations occupy 41.91–44.96% (53.45–57.44%) for directly adjacent cases, and 58.06–61.65% (35.86–40.67%) for indirectly adjacent cases, respectively.

### Spatial partial (SP) correlations of link travel speeds

As Fig. 4 shows, the two road networks lead to quite similar SP correlations and different time ranges have small effects on the corresponding results. For the two networks, the majority of SP correlations are insignificant, specifically 69.43–76.42% for directly adjacent cases and 88.24–89.18% for indirectly adjacent cases. Significant positive SP correlations only cover 21.08–27.72% and 4.7–7.09% respectively for directly and indirectly adjacent cases. This shows that it is not easy to obtain the travel speed on a link based on the speeds on its neighboring links and their spatial and SP correlations, which is inconsistent with Esfahani and Gayah’s finding^28^. A possible reason is that their study is based on a road network with only 40 links.

### Spatial–temporal (ST) correlations and spatial–temporal partial (STP) correlations of link travel speeds

**Figure 5:**
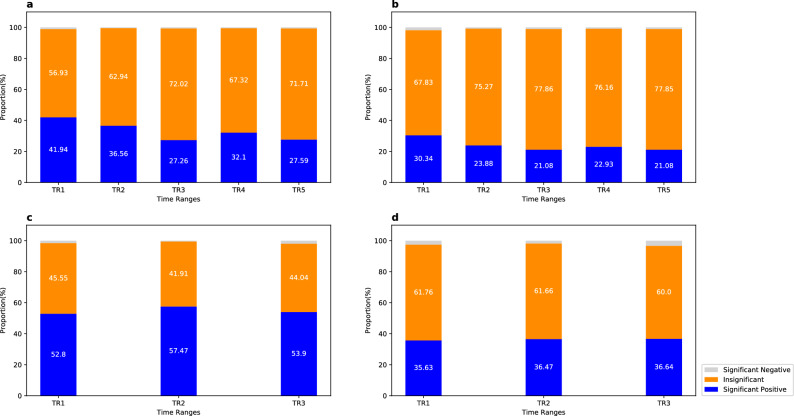
Proportions of Spatial–temporal (ST) correlations and spatial–temporal partial (STP) correlations of travel speeds. We provide the average results of different time periods here because different time periods have little effect on ST and STP correlation (see Supplemental Tables S6 and S7). The three colors in each bar represent the average proportions from different time periods within a specific time range, for significant positive, insignificant, and significant negative spatial or SP correlations, respectively. (**a**) Urban road network (ST). (**b**) Urban road network (STP). (**c**) Freeway network (ST). (**d**) Freeway network (STP).

As Fig. 5 shows, for the urban road network, less than 1.13% (1.83%) of ST (STP) correlations are significantly negative; while 27.26–41.94% (21.08–30.34%) of ST (STP) correlations are significantly positive. Comparing to the urban road network, more significant positive ST (STP) correlations can be observed in the freeway network, which range within [52.8%, 57.47%] ([35.63%, 36.64%]).

## Discussion

### Marginal distributions and correlations on different types of road links

In urban road networks, there exist different types of road links in terms of different traffic capacities and design speeds, such as primary, secondary, tertiary and residential. We further investigate if and how different road types exhibit different marginal distributions and correlations. Different road link types produce quite similar distributions. For different types of road links in the urban road network (Supplemental Table S1), 90.14–96.23% of link travel speeds are normally distributed, while the lognormal, the Gumbel, the beta, and the Weibull distributions cover 2.58–4.34%, 0.38–3.02%, 0.22–1.67% and 0.01–0.02%, respectively. To measure the effects of different road types on correlations, given a specific time period lag and time range, we obtain their percentage difference on the two road networks for each correlation type (i.e., significant positive, insignificant, or significant negative), and then calculate the mean and standard deviation of the corresponding differences. A smaller mean and standard deviation imply lower effects by road network type. For the four types of road links considered, we have 6 link type pairs for comparison and can get the range of the mean and standard deviation of percentage differences for each pair of link types (See Supplemental Table S8). Different road link types have small effects on the various correlations. Taking ST and STP correlations as examples, the corresponding mean (standard deviation) for different link type pairs range within [0.07%, 0.31%] ([0.04%, 0.10%]), [0.73%, 3.06%] ([0.37%, 1.92%]), and [0.90%, 3.02%] ([0.42%, 1.95%]).

Our findings have some practical implications. First, the new knowledge discovered about distributions and correlations can be used to generate more realistic simulated data of link travel speeds in road networks, and drive the research in logistics, transportation, and traffic management under uncertain environments. Second, the urban road network used is a large-scale ring-radial network. This network and its sub-networks exist widely in the real world, which makes our findings representative. Third, comparing with correlations of link travel speeds, it is better to use the partial correlations to reflect the real correlations of two travel speeds since they remove the effects of speeds from neighboring links and preceding periods. This research faces some limitations due to the limitations of the travel speed datasets used. For example, we cannot consider the effects of viaducts and tunnels in the road network, and the effects of traffic signal controls on link travel speeds and the resulting marginal distributions and correlations. Our future work will address these issues. Moreover, this research considers marginal distributions and the Pearson correlation only and future work can further consider joint and conditional distributions, and other correlations such as Spearman correlations and multiple correlations. Our future work can also investigate the autocorrelations of travel speeds based on autocorrelation statistics such as Moran’s I^30^ and Getis-Ord general G^31^.

In closing, this paper empirically analyzes for the first time the marginal distributions and various correlations of link travel speeds in a large-scale urban road network and a freeway networks based on large volumes of link travel speed data. The new knowledge does not reveal all facts about distributions and correlations of link travel speeds in real-world road networks, but it is crucial for meeting the requirements of travel speed data and facilitating research in logistics, transportation and traffic management.

## Methods

### Road network and travel speeds data

In the urban road network shown in Fig. 1a, we classify the road links into four types based on the link types specified in the Volunteered Geographic Information (VGI) site OpenStreetMaps (OSM), including primary, secondary, tertiary, residential and unclassified. Considering the actual road and traffic features, the primary links in Fig. 1a consist of primary, motorway and trunk roads in OSM. We collect a total of 5.5 billion raw GPS trajectory samples produced by more than 12,000 floating taxis as source data from Chengdu, a megacity in Western China. The road network contains 1,902 nodes and 5,943 directed links. The GPS trajectory data from all floating taxis are collected from June 1 to September 17, 2017. Based on these raw trajectory data, we obtain a 110-day link travel speed dataset with 300 2-min time periods according to Guo et al.’s method^7^. The 300 time periods are from 5 representative 2-h time ranges, including 3:00–5:00, 8:00–10:00, 12:00–14:00, 17:00–19:00, and 21:00–23:00. There is a total of 196,119,000 speed records.

We collect all speed readings of the 3,417 speed detection stations of the California freeway network via the Caltrans Performance Measurement System (PeMS), from May 1 to September 22 in 2017, excluding weekends and holidays. The freeway network, as shown in Fig. 1b, contains 168 nodes and 438 directed links. Each link travel speed in each time period is obtained by averaging the speed values from all detection stations on this link in this period. Each period is 5 min. We collect the link travel speeds of three representative 2-h time ranges; 8:00–10:00, 12:00–14:00 and 17:00–19:00. We finally obtain a 102-day dataset with a total of 3,216,672 road travel link speeds from 72.5-min time periods.

### Distributions

On the basis of the one-sample Kolmogorov-Smirnov (K-S) test^32^ with a significance level of 0.05, we use different single-mode probability distributions to fit the set of travel speeds on each road link. The candidate distributions include normal, lognormal, Gumbel, beta, Weibull, gamma, exponential, Generalized Pareto, and Burr. We select out the distributions that can fit more than 0.1% of travel speed variables in either the urban road or the freeway network. Five distributions end up being used: normal, lognormal, Gumbel, beta and Weibull.

### Correlations

We investigate three commonly used correlations of travel speeds; temporal correlations, spatial correlations, temporal and spatial correlations, and their partial correlations. The partial correlation is a measure of the association degree of two random variables, with the effect of a set of controlling random variables removed. All correlations are calculated according to the Pearson correlation coefficients.

The correlation between travel speeds on a certain link in two different time periods is called a temporal correlation. The correlation of travel speeds on two different links in a certain time period is called as spatial correlation. The correlation of travel speeds on two different links in two different time periods is called a spatial–temporal correlation. According to the Student's t-test at the significance level of 5%, we consider two variables of travel speeds as significantly correlated at a confidence level of 95% if the $p$ value of t-statistic is less than or equal to 0.05; otherwise, they are insignificantly correlated. Two speeds are significantly correlated at $\left| r \right| \ge 0.1874$ for the urban road network and at $\left| r \right| \ge 0.1946$ for the freeway network.

### Temporal correlations and temporal partial correlations

The temporal correlation is the correlation of travel speeds on a certain link in two different time periods. The larger the two periods’ time lag, the less effect the earlier speed has on the latter. Among these correlations, we calculate the percentages of significant positive, insignificant, and significant negative correlations.

To calculate the temporal partial correlations of travel speeds in period $i$ and period $i +k$ on a given link, we take the speeds between period $i + 1$ and period $i + k - 1$ as the controlling variables. The temporal partial correlation between two consecutive periods equals its corresponding temporal correlation.

### Spatial correlations and spatial partial correlations

Spatial correlations are defined as the correlations of travel speeds on two different links in a given time period. The larger the two links’ distance is, the less effects the earlier speed has on the latter. We consider the spatial correlations of two links based on two cases, directly adjacent and indirectly adjacent. Two links are directly adjacent if they share a node and have the same traffic-flow direction. They are indirectly adjacent if the two links share a node but have different traffic-flow directions, or the two links have the same traffic-flow direction and connect to different nodes of the same link.

The travel speed on a link is not affected by the speeds on links far away from the link. For a given link $l$, we define link $l^{\prime}$ as a nearby link if the distance between the midpoints of link $l$ and link $l^{\prime}$ is not larger than 1 km. To calculate the spatial partial correlations of link $l$ and link $l^{\prime}$ in a period, we take the travel speeds on the nearby links of each link as the controlling variables.

### Spatial–temporal correlations and spatial–temporal partial correlations

Both temporal correlations and spatial correlations are special cases of spatial–temporal correlations, which are defined as the correlations of travel speeds on two different links in two different time periods. Compared to the spatial correlation defined earlier, the differences of spatial–temporal correlation calculation include (1) considering two consecutive time periods, and (2) considering directly adjacent cases only. We calculate the spatial–temporal partial correlation based on the spatial partial correlation. The only difference is that we consider the speeds in two consecutive time periods here instead of considering speeds in the same period.

## Supplementary information

Supplementary information.
