# Supplementary material for: Understanding the marginal distributions and correlations of link travel speeds in road networks
Source: Sci Rep. 2020 Jul 16;10:11821. doi: 10.1038/s41598-020-68810-9 (PMC7366716; doi:10.1038/s41598-020-68810-9)
Supplement: Supplementary file 1 — Supplementary information. [file 41598_2020_68810_MOESM1_ESM.pdf]

Supplementary Information

Understanding the marginal distributions and correlations of link travel speeds in road networks

Feng Guo[1], Xin Gu[1], Zhaoxia Guo[1,*], Yucheng Dong[1,*] and Stein W. Wallace[1,2] [1]Business School, Sichuan University, Chengdu 610065, China
[2]NHH Norwegian School of Economics, Bergen, Norway

*emails: zx.guo@alumni.polyu.edu.hk, ycdong@scu.edu.cn

List of Supplementary Tables

Table S1: Proportions of different distribution types. This table presents the results for different road network types, different road link types and different time ranges.

Table S2: Proportions of different temporal correlations of travel speeds between period i and period $i+k$ $(k=1,2,3,4,5)$. k denotes the time period lag. The five (three) elements in each cell represent the correlations of five (three) different time ranges in the urban road (freeway) network.

Table S3: Proportions of different temporal partial correlations of travel speeds between period i and period $i+k$ $(k=2,3,4,5)$. The five (three) elements in each cell represent the correlations of five (three) different time ranges in the urban road (freeway) network.

Table S4: Proportions of different spatial correlations of travel speeds in different time ranges/periods for both directly and indirectly adjacent cases. The columns "directly" and "indirectly" present the results of directly and indirectly adjacent cases. The six elements in each cell represent the spatial correlations from 6 different time periods, respectively.

Table S5: Proportions of different spatial partial correlations of travel speeds in different time ranges/periods for both directly and indirectly adjacent cases. The columns "directly" and "indirectly" present the results of directly and indirectly adjacent cases. The six elements in each cell represent the spatial partial correlations from 6 different time periods, respectively.

Table S6: Proportions of Spatial-temporal correlations of travel speeds.

Table S7: Proportions of Spatial-temporal partial correlations of travel speeds.

Table S8: Differences in proportions of different types of correlations between different types of road links. Given a correlation type (temporal, temporal partial, etc.) and a road link (primary, secondary, tertiary, and residential), we obtain its proportions of significant positive, insignificant, and significant negative correlations in different time ranges and time periods. This table presents the mean and the standard deviation of differences of these proportions between any two types of road links. For example, the 0.08 (0.11) marked in yellow represents that, for the temporal correlation, the mean (standard deviation) of differences of the proportions of significant negative correlations between primary and secondary links is 0.08% (0.11%).

Table S1. Proportions of different distribution types. This table presents the results for different road network types, different road link types and different time ranges.

Road network type	Road link type	Time ranges	Normal (%)	Lognormal (%)	Gumbel (%)	Beta (%)	Weibull (%)	None of above (%)
Urban road network	All	3-5am	95.09	1.77	1.36	0.63	0.01	1.14
		8-10am	92.59	4.69	1.35	1	0.02	0.35
		12-14am	94.07	3.09	1.68	0.77	0.02	0.37
		5-7pm	92.7	4.42	1.42	1.03	0.01	0.42
		9-11pm	94.62	2.81	1.65	0.6	0.01	0.31
		All	93.81	3.36	1.49	0.81	0.01	0.52
	Primary	All	90.14	4.34	3.02	1.67	0.02	0.81
	Secondary	All	95.52	2.78	0.95	0.38	0.01	0.36
	Tertiary	All	95.54	2.58	0.82	0.55	0.01	0.5
	Residential	All	96.23	2.72	0.38	0.22	0.02	0.43
Freeway network	All	8-10am	58.24	6.14	23.53	6.88	0.1	5.11
		12-14am	57.71	1.12	29.14	5.26	0.1	6.67
		5-7pm	57.1	9.6	19.27	5.96	0.19	7.88
		All	57.68	5.62	23.98	6.03	0.14	6.55

Table S2. Proportions of different temporal correlations of travel speeds between period i and period $i+k$ (k=1,2,3,4,5). k denotes the time period lag. The five (three) elements in each cell represent the correlations of five (three) different time ranges in the urban road (freeway) network.

Road network type	Road type	k	Significant negative(%)	Insignificant(%)	Significant positive(%)
Urban road network	All	1	[0.1, 0.02, 0.03, 0.02, 0.02]	[2.15, 4.07, 8.19, 5.54, 7.13]	[97.75, 95.91, 91.78, 94.44, 92.85]
		2	[0.29, 0.04, 0.06, 0.05, 0.06]	[3.84, 6.08, 11.0, 7.73, 10.25]	[95.87, 93.88, 88.94, 92.22, 89.69]
		3	[0.66, 0.13, 0.23, 0.16, 0.22]	[33.72, 32.55, 44.49, 36.42, 43.09]	[65.62, 67.32, 55.28, 63.42, 56.69]
		4	[1.28, 0.28, 0.42, 0.3, 0.44]	[68.09, 53.0, 68.55, 57.59, 67.32]	[30.63, 46.72, 31.03, 42.12, 32.24]
		5	[3.38, 0.82, 1.26, 0.85, 1.32]	[89.02, 70.28, 85.1, 74.75, 84.4]	[7.6, 28.9, 13.64, 24.4, 14.28]
	Primary	1	[0.07, 0.01, 0.04, 0.03, 0.04]	[3.83, 5.59, 12.2, 7.63, 10.73]	[96.1, 94.4, 87.76, 92.34, 89.23]
		2	[0.21, 0.03, 0.06, 0.06, 0.09]	[5.89, 7.61, 15.14, 9.81, 14.51]	[93.9, 92.36, 84.8, 90.13, 85.4]
		3	[0.54, 0.13, 0.25, 0.19, 0.3]	[39.03, 28.52, 44.44, 32.98, 43.22]	[60.43, 71.35, 55.31, 66.83, 56.48]
		4	[1.07, 0.21, 0.42, 0.29, 0.53]	[70.23, 45.32, 65.69, 49.96, 64.1]	[28.7, 54.47, 33.89, 49.75, 35.37]
		5	[2.9, 0.62, 1.15, 0.7, 1.2]	[89.46, 61.08, 80.98, 65.67, 79.87]	[7.64, 38.3, 17.87, 33.63, 18.93]
	Secondary	1	[0.1, 0.02, 0.04, 0.02, 0.02]	[2.21, 4.95, 9.7, 6.55, 8.22]	[97.69, 95.03, 90.26, 93.43, 91.76]
		2	[0.31, 0.08, 0.08, 0.04, 0.07]	[3.86, 7.27, 12.72, 8.9, 11.72]	[95.83, 92.65, 87.2, 91.06, 88.21]
		3	[0.66, 0.18, 0.25, 0.2, 0.24]	[34.83, 34.44, 47.46, 38.65, 45.68]	[64.51, 65.38, 52.29, 61.15, 54.08]
		4	[1.26, 0.29, 0.47, 0.38, 0.44]	[68.77, 53.44, 70.21, 58.24, 68.73]	[29.97, 46.27, 29.32, 41.38, 30.83]
		5	[3.44, 0.79, 1.23, 0.88, 1.28]	[89.32, 69.93, 86.44, 74.1, 85.09]	[7.24, 29.28, 12.33, 25.02, 13.63]
	Tertiary	1	[0.15, 0.04, 0.04, 0.03, 0.03]	[0.83, 2.61, 4.3, 3.36, 3.89]	[99.02, 97.35, 95.66, 96.61, 96.08]
		2	[0.4, 0.09, 0.09, 0.07, 0.08]	[2.23, 4.36, 6.66, 5.29, 6.44]	[97.37, 95.55, 93.25, 94.64, 93.48]
		3	[0.84, 0.22, 0.28, 0.19, 0.29]	[28.55, 34.94, 42.94, 37.6, 42.1]	[70.61, 64.84, 56.78, 62.21, 57.61]
		4	[1.66, 0.46, 0.6, 0.39, 0.58]	[65.19, 58.77, 69.86, 62.04, 69.06]	[33.15, 40.77, 29.54, 37.57, 30.36]
		5	[4.06, 1.28, 1.7, 1.26, 1.75]	[87.93, 77.39, 87.81, 80.53, 87.25]	[8.01, 21.33, 10.49, 18.21, 11.0]
	Residential	1	[0.14, 0.01, 0.01, 0.01, 0.0]	[0.8, 1.61, 3.36, 2.56, 2.82]	[99.06, 98.38, 96.63, 97.43, 97.18]
		2	[0.36, 0.02, 0.05, 0.02, 0.01]	[2.1, 3.24, 5.44, 4.42, 5.14]	[97.54, 96.74, 94.51, 95.56, 94.85]
		3	[0.81, 0.08, 0.16, 0.1, 0.13]	[27.99, 32.62, 40.36, 36.29, 40.36]	[71.2, 67.3, 59.48, 63.61, 59.51]

		4	[1.51, 0.23, 0.31, 0.25, 0.35]	[65.86, 57.94, 68.74, 62.81, 69.77]	[32.63, 41.83, 30.95, 36.94, 29.88]
		5	[3.85, 0.9, 1.3, 0.95, 1.51]	[88.34, 77.9, 87.72, 82.38, 88.65]	[7.81, 21.2, 10.98, 16.67, 9.84]
Freeway network	All	1	[0.0, 0.0, 0.0]	[0.01, 0.01, 0.0]	[99.99, 99.99, 100.0]
		2	[0.0, 0.0, 0.0]	[0.02, 0.02, 0.07]	[99.98, 99.98, 99.93]
		3	[0.0, 0.0, 0.0]	[0.16, 0.08, 0.06]	[99.84, 99.92, 99.94]
		4	[0.0, 0.0, 0.0]	[0.31, 0.24, 0.13]	[99.69, 99.76, 99.87]
		5	[0.0, 0.0, 0.0]	[0.59, 0.66, 0.37]	[99.41, 99.34, 99.63]

Table S3. Proportions of different temporal partial correlations of travel speeds between period i and period $i+k$ (k =2,3,4,5). The five (three) elements in each cell represent the correlations of five (three) different time ranges in the urban road (freeway) network.

Road network type	Road type	*k*	Significant Negative(%)	Insignificant(%)	Significant Positive(%)
Urban road network	Total	2	[1.61, 0.27, 0.36, 0.3, 0.41]	[81.92, 64.24, 69.53, 65.13, 70.11]	[16.47, 35.49, 30.11, 34.57, 29.48]
		3	[21.56, 13.18, 15.83, 14.15, 15.26]	[77.7, 84.69, 82.45, 83.55, 83.07]	[0.74, 2.13, 1.72, 2.3, 1.67]
		4	[2.2, 1.01, 1.28, 1.02, 1.36]	[96.54, 96.69, 96.74, 96.6, 96.62]	[1.26, 2.3, 1.98, 2.38, 2.02]
		5	[2.63, 0.91, 1.23, 1.0, 1.26]	[96.3, 96.53, 96.39, 96.3, 96.51]	[1.07, 2.56, 2.38, 2.7, 2.23]
	Primary	2	[1.3, 0.22, 0.34, 0.3, 0.42]	[79.06, 62.56, 68.67, 62.91, 69.0]	[19.64, 37.22, 30.99, 36.79, 30.58]
		3	[20.43, 9.3, 12.21, 10.25, 11.47]	[78.9, 87.34, 84.84, 85.78, 85.52]	[0.67, 3.36, 2.95, 3.97, 3.01]
		4	[2.1, 0.9, 1.28, 0.99, 1.37]	[96.65, 96.19, 96.13, 95.73, 95.82]	[1.25, 2.91, 2.59, 3.28, 2.81]
		5	[2.28, 0.83, 1.3, 0.96, 1.32]	[96.46, 96.25, 95.83, 95.85, 95.79]	[1.26, 2.92, 2.87, 3.19, 2.89]
	Secondary	2	[1.3, 0.27, 0.34, 0.3, 0.39]	[82.02, 62.39, 68.98, 64.01, 69.3]	[16.68, 37.34, 30.68, 35.69, 30.31]
		3	[22.77, 12.87, 15.83, 14.04, 15.28]	[76.55, 84.76, 82.57, 83.49, 83.13]	[0.68, 2.37, 1.6, 2.47, 1.59]
		4	[1.95, 0.98, 1.31, 1.03, 1.37]	[96.85, 96.53, 96.69, 96.5, 96.63]	[1.2, 2.49, 2.0, 2.47, 2.0]
		5	[2.47, 0.86, 1.17, 1.06, 1.29]	[96.4, 96.37, 96.36, 95.98, 96.48]	[1.13, 2.77, 2.47, 2.96, 2.23]
	Tertiary	2	[2.14, 0.46, 0.61, 0.45, 0.6]	[84.13, 66.12, 70.34, 66.73, 71.33]	[13.73, 33.42, 29.05, 32.82, 28.07]
		3	[22.61, 17.44, 19.89, 18.1, 19.26]	[76.52, 81.53, 79.29, 80.92, 80.01]	[0.87, 1.03, 0.82, 0.98, 0.73]
		4	[2.49, 1.27, 1.45, 1.19, 1.54]	[96.19, 97.03, 97.06, 97.06, 97.0]	[1.32, 1.7, 1.49, 1.75, 1.46]
		5	[3.11, 1.2, 1.39, 1.19, 1.44]	[95.97, 96.61, 96.72, 96.52, 96.73]	[0.92, 2.19, 1.89, 2.29, 1.83]
	Residential	2	[2.06, 0.29, 0.35, 0.28, 0.42]	[85.25, 68.24, 72.18, 68.97, 73.34]	[12.69, 31.47, 27.47, 30.75, 26.24]
		3	[21.22, 16.7, 19.08, 17.61, 19.25]	[77.91, 82.37, 80.2, 81.54, 80.19]	[0.87, 0.93, 0.72, 0.85, 0.56]
		4	[2.47, 1.02, 1.13, 1.04, 1.26]	[96.21, 97.39, 97.52, 97.5, 97.54]	[1.32, 1.59, 1.35, 1.46, 1.2]
		5	[3.08, 0.92, 1.14, 1.03, 1.22]	[96.02, 97.18, 97.07, 97.11, 97.3]	[0.9, 1.9, 1.79, 1.86, 1.48]
Freeway network	ALL	2	[2.97, 1.96, 2.36]	[95.79, 97.33, 97.2]	[1.24, 0.71, 0.44]
		3	[0.25, 0.11, 0.07]	[98.74, 99.27, 99.36]	[1.01, 0.62, 0.57]
		4	[0.14, 0.02, 0.1]	[98.8, 99.56, 98.95]	[1.06, 0.42, 0.95]
		5	[0.24, 0.02, 0.36]	[99.41, 99.76, 99.53]	[0.35, 0.22, 0.11]

Table S4. Proportions of different spatial correlations of travel speeds in different time ranges/periods for both directly and indirectly adjacent cases. The columns "directly" and "indirectly" present the results of directly and indirectly adjacent cases. The six elements in each cell represent the spatial correlations from 6 different time periods, respectively.

Road network type	Road type	Time ranges	Significant negative(%)		Insignificant(%)		Significant positive(%)	
			directly	indirectly	directly	indirectly	directly	indirectly
	Total	3-5am	[0.97, 0.8, 0.87, 1.12, 1.01, 1.04]	[1.73, 1.57, 1.68, 1.79, 1.71, 1.72]	[45.56, 44.39, 43.04, 41.37, 41.99, 40.65]	[83.78, 83.16, 82.04, 80.44, 80.44, 80.39]	[53.47, 54.81, 56.09, 57.51, 57.0, 58.31]	[14.49, 15.27, 16.28, 17.77, 17.85, 17.89]
		8-10am	[0.16, 0.27, 0.33, 0.37, 0.4, 0.46]	[0.62, 0.88, 1.01, 1.15, 1.23, 1.37]	[41.7, 46.46, 49.23, 54.65, 56.18, 56.08]	[71.74, 76.85, 80.75, 84.84, 86.72, 87.16]	[58.14, 53.27, 50.44, 44.98, 43.42, 43.46]	[27.64, 22.27, 18.24, 14.01, 12.05, 11.47]
		12-14am	[0.47, 0.63, 0.48, 0.49, 0.47, 0.51]	[1.34, 1.42, 1.41, 1.59, 1.43, 1.4]	[58.19, 58.35, 58.7, 59.25, 58.53, 58.77]	[88.73, 89.39, 89.38, 89.3, 89.32, 88.75]	[41.34, 41.02, 40.82, 40.26, 41.0, 40.72]	[9.93, 9.19, 9.21, 9.11, 9.25, 9.85]
		5-7pm	[0.48, 0.49, 0.42, 0.45, 0.37, 0.43]	[1.24, 1.31, 1.27, 1.24, 1.13, 1.18]	[56.21, 55.21, 53.75, 53.53, 54.38, 55.93]	[86.93, 86.1, 84.5, 84.82, 85.63, 86.82]	[43.31, 44.3, 45.83, 46.02, 45.25, 43.64]	[11.83, 12.59, 14.23, 13.94, 13.24, 12.0]
		9-11pm	[0.63, 0.52, 0.42, 0.52, 0.43, 0.51]	[1.35, 1.43, 1.43, 1.55, 1.44, 1.42]	[59.53, 57.75, 57.14, 57.49, 57.81, 60.09]	[89.16, 88.98, 88.1, 88.19, 88.13, 89.2]	[39.84, 41.73, 42.44, 41.99, 41.76, 39.4]	[9.49, 9.59, 10.47, 10.26, 10.43, 9.38]
	Primary	3-5am	[1.22, 0.64, 0.87, 1.07, 0.91, 0.89]	[1.87, 1.63, 1.76, 1.87, 1.68, 1.89]	[46.56, 46.71, 44.39, 42.83, 43.78, 42.38]	[84.05, 83.83, 82.34, 81.38, 81.6, 81.38]	[52.22, 52.65, 54.74, 56.1, 55.31, 56.73]	[14.08, 14.54, 15.9, 16.75, 16.72, 16.73]
		8-10am	[0.2, 0.31, 0.35, 0.39, 0.47, 0.47]	[0.74, 1.03, 1.04, 1.1, 1.32, 1.47]	[41.59, 45.35, 47.62, 52.87, 54.89, 54.53]	[71.09, 74.79, 78.74, 83.04, 85.29, 85.93]	[58.21, 54.34, 52.03, 46.74, 44.64, 45.0]	[28.17, 24.18, 20.22, 15.86, 13.39, 12.6]
		12-14am	[0.39, 0.88, 0.48, 0.52, 0.68, 0.52]	[1.43, 1.42, 1.53, 1.6, 1.56, 1.47]	[57.88, 57.7, 58.91, 59.73, 58.65, 59.21]	[88.1, 88.9, 88.8, 88.83, 88.71, 88.18]	[41.73, 41.42, 40.61, 39.75, 40.67, 40.27]	[10.47, 9.68, 9.67, 9.57, 9.73, 10.35]
		5-7pm	[0.62, 0.62, 0.57, 0.57, 0.42, 0.61]	[1.47, 1.48, 1.47, 1.4, 1.19, 1.21]	[56.35, 54.83, 53.31, 52.25, 52.15, 53.97]	[86.22, 85.13, 83.24, 82.9, 83.1, 84.46]	[43.03, 44.55, 46.12, 47.18, 47.43, 45.42]	[12.31, 13.39, 15.29, 15.7, 15.71, 14.33]
		9-11pm	[0.66, 0.62, 0.4, 0.71, 0.53, 0.54]	[1.57, 1.64, 1.66, 1.75, 1.65, 1.5]	[60.48, 58.01, 57.33, 58.04, 58.73, 60.72]	[88.43, 88.43, 87.16, 87.41, 87.54, 88.42]	[38.86, 41.37, 42.27, 41.25, 40.74, 38.74]	[10.0, 9.93, 11.18, 10.84, 10.81, 10.08]

Urban road network	Secondary	3-5am	[0.83, 0.55, 0.57, 1.07, 1.01, 0.83]	[1.73, 1.38, 1.69, 1.68, 1.65, 1.41]	[44.71, 42.52, 41.87, 39.04, 39.73, 39.76]	[82.67, 81.86, 80.39, 79.25, 79.09, 79.79]	[54.46, 56.93, 57.56, 59.89, 59.26, 59.41]	[15.6, 16.76, 17.92, 19.07, 19.26, 18.8]
		8-10am	[0.1, 0.14, 0.39, 0.32, 0.32, 0.57]	[0.59, 0.79, 1.0, 1.34, 0.99, 1.35]	[42.16, 46.23, 49.81, 54.07, 55.47, 55.57]	[72.38, 76.32, 80.2, 83.96, 86.13, 86.49]	[57.74, 53.63, 49.8, 45.61, 44.21, 43.86]	[27.03, 22.89, 18.8, 14.7, 12.88, 12.16]
		12-14am	[0.71, 0.41, 0.24, 0.51, 0.34, 0.65]	[1.51, 1.49, 1.24, 1.51, 1.27, 1.51]	[58.92, 58.74, 59.59, 60.64, 59.18, 58.9]	[88.66, 89.15, 89.67, 89.32, 89.5, 89.01]	[40.37, 40.85, 40.17, 38.85, 40.48, 40.45]	[9.83, 9.36, 9.09, 9.17, 9.23, 9.48]
		5-7pm	[0.39, 0.53, 0.45, 0.22, 0.51, 0.59]	[1.28, 1.6, 1.41, 1.17, 1.23, 1.28]	[55.78, 55.77, 53.65, 53.67, 54.76, 56.87]	[86.26, 85.52, 83.44, 84.44, 85.02, 87.11]	[43.83, 43.7, 45.9, 46.11, 44.73, 42.54]	[12.46, 12.88, 15.15, 14.39, 13.75, 11.61]
		9-11pm	[0.83, 0.59, 0.24, 0.36, 0.43, 0.32]	[1.16, 1.26, 1.35, 1.53, 1.51, 1.38]	[59.44, 57.82, 57.48, 57.44, 56.57, 60.17]	[89.56, 88.42, 87.61, 87.4, 87.33, 88.69]	[39.73, 41.59, 42.28, 42.2, 43.0, 39.51]	[9.28, 10.32, 11.04, 11.07, 11.16, 9.93]
	Tertiary	3-5am	[0.89, 1.11, 1.08, 1.18, 1.06, 1.46]	[1.69, 1.7, 1.75, 1.92, 1.57, 1.89]	[41.78, 41.45, 41.48, 39.27, 39.94, 37.41]	[81.98, 81.76, 80.88, 78.49, 79.42, 78.42]	[57.33, 57.44, 57.44, 59.55, 59.0, 61.13]	[16.33, 16.54, 17.37, 19.59, 19.01, 19.69]
		8-10am	[0.19, 0.41, 0.38, 0.48, 0.41, 0.54]	[0.61, 0.91, 1.17, 1.25, 1.52, 1.45]	[42.51, 47.51, 50.99, 55.93, 57.12, 56.36]	[74.17, 79.42, 82.53, 86.35, 87.87, 88.18]	[57.3, 52.08, 48.63, 43.59, 42.47, 43.1]	[25.22, 19.67, 16.3, 12.4, 10.61, 10.37]
		12-14am	[0.27, 0.68, 0.67, 0.44, 0.43, 0.71]	[1.29, 1.6, 1.54, 1.65, 1.35, 1.45]	[56.69, 57.58, 56.96, 56.58, 56.57, 56.52]	[88.97, 89.24, 89.04, 89.1, 89.32, 88.45]	[43.04, 41.74, 42.37, 42.98, 43.0, 42.77]	[9.74, 9.16, 9.42, 9.25, 9.33, 10.1]
		5-7pm	[0.41, 0.57, 0.41, 0.62, 0.32, 0.33]	[1.17, 1.2, 1.43, 1.37, 1.32, 1.3]	[55.6, 53.84, 54.49, 53.69, 55.07, 56.93]	[87.55, 86.53, 85.82, 85.94, 87.19, 87.77]	[43.99, 45.59, 45.1, 45.69, 44.61, 42.74]	[11.28, 12.27, 12.75, 12.69, 11.49, 10.93]
		9-11pm	[0.64, 0.43, 0.6, 0.57, 0.52, 0.56]	[1.19, 1.41, 1.36, 1.47, 1.4, 1.41]	[57.46, 55.79, 55.28, 55.5, 56.79, 57.49]	[88.52, 88.8, 88.15, 87.95, 87.81, 89.06]	[41.9, 43.78, 44.12, 43.93, 42.69, 41.95]	[10.29, 9.79, 10.49, 10.58, 10.79, 9.54]
		3-5am	[1.05, 1.05, 1.06, 1.52, 1.12, 1.25]	[1.64, 1.78, 1.68, 1.83, 1.78, 1.85]	[43.23, 41.14, 40.08, 38.6, 39.64, 38.77]	[82.98, 81.22, 80.62, 78.66, 77.91, 77.72]	[55.72, 57.81, 58.86, 59.88, 59.24, 59.98]	[15.38, 17.0, 17.7, 19.51, 20.31, 20.43]
		8-10am	[0.11, 0.29, 0.21, 0.27, 0.29, 0.36]	[0.45, 0.77, 0.94, 1.02, 1.1, 1.12]	[38.07, 44.64, 47.11, 53.55, 53.19, 53.55]	[70.12, 78.67, 82.56, 85.83, 87.08, 87.46]	[61.82, 55.07, 52.68, 46.18, 46.52, 46.09]	[29.42, 20.56, 16.5, 13.15, 11.82, 11.42]

	Residential	12-14am	[0.53, 0.55, 0.57, 0.44, 0.21, 0.29]	[1.18, 1.2, 1.29, 1.39, 1.37, 1.24]	[55.48, 56.58, 54.87, 55.66, 55.23, 55.21]	[88.46, 89.55, 89.35, 89.24, 89.23, 88.63]	[43.99, 42.87, 44.56, 43.9, 44.56, 44.5]	[10.36, 9.25, 9.36, 9.37, 9.4, 10.13]
		5-7pm	[0.48, 0.21, 0.21, 0.46, 0.32, 0.36]	[1.04, 0.92, 0.96, 1.0, 0.86, 1.02]	[52.74, 53.0, 51.35, 51.74, 53.74, 54.18]	[87.35, 86.94, 85.23, 86.3, 87.7, 88.25]	[46.78, 46.79, 48.44, 47.8, 45.94, 45.46]	[11.61, 12.14, 13.81, 12.7, 11.44, 10.73]
		9-11pm	[0.59, 0.36, 0.51, 0.4, 0.32, 0.48]	[1.24, 1.23, 1.27, 1.36, 1.1, 1.35]	[56.65, 55.25, 54.1, 54.81, 54.31, 57.16]	[90.07, 89.31, 89.03, 89.52, 88.87, 90.21]	[42.76, 44.39, 45.39, 44.79, 45.37, 42.36]	[8.69, 9.46, 9.7, 9.12, 10.03, 8.44]
Freeway network	ALL	8-10am	[2.93, 1.71, 1.59, 1.22, 0.98, 1.1]	[3.4, 3.33, 1.63, 2.45, 1.56, 1.56]	[40.85, 42.44, 42.8, 46.1, 48.41, 49.15]	[57.34, 55.91, 59.04, 64.13, 66.17, 67.32]	[56.22, 55.85, 55.61, 52.68, 50.61, 49.75]	[39.26, 40.76, 39.33, 33.42, 32.27, 31.12]
		12-14am	[0.49, 0.24, 0.85, 0.85, 0.73, 0.73]	[1.02, 1.43, 1.29, 1.36, 1.09, 1.43]	[43.29, 43.05, 41.95, 39.63, 42.32, 41.22]	[60.67, 59.31, 58.97, 57.13, 56.39, 55.91]	[56.22, 56.71, 57.2, 59.51, 56.95, 58.05]	[38.31, 39.26, 39.74, 41.51, 42.52, 42.66]
		5-7pm	[1.95, 2.32, 2.2, 2.2, 2.44, 1.34]	[3.13, 3.4, 3.6, 3.33, 2.45, 2.04]	[45.12, 44.39, 44.51, 46.1, 44.02, 40.24]	[62.77, 62.43, 61.35, 61.41, 61.21, 57.74]	[52.93, 53.29, 53.29, 51.7, 53.54, 58.42]	[34.1, 34.17, 35.05, 35.26, 36.34, 40.22]

Table S5. Proportions of different spatial partial correlations of travel speeds in different time ranges/periods for both directly and indirectly adjacent cases. The columns "directly" and "indirectly" present the results of directly and indirectly adjacent cases. The six elements in each cell represent the spatial partial correlations from 6 different time periods, respectively.

Road network type	Road type	Time ranges	Significant negative(%)		Insignificant(%)		Significant positive(%)	
			directly	indirectly	directly	indirectly	directly	indirectly
	All	3-5am	[2.66, 2.63, 2.98, 2.92, 2.96, 3.12]	[5.8, 5.71, 5.5, 5.36, 5.29, 5.33]	[74.68, 74.83, 74.74, 74.35, 75.02, 74.73]	[88.11, 88.51, 88.37, 88.29, 88.16, 88.33]	[22.66, 22.54, 22.28, 22.73, 22.02, 22.15]	[6.09, 5.78, 6.13, 6.35, 6.55, 6.34]
		8-10am	[2.62, 2.42, 2.46, 2.33, 2.23, 2.41]	[6.22, 6.11, 6.44, 6.2, 6.21, 6.17]	[76.29, 76.51, 76.17, 76.87, 76.32, 76.12]	[88.34, 88.55, 88.33, 89.07, 89.12, 89.16]	[21.09, 21.07, 21.37, 20.8, 21.45, 21.47]	[5.44, 5.34, 5.23, 4.73, 4.67, 4.67]
		12-14am	[2.57, 2.33, 2.29, 2.5, 2.17, 2.32]	[6.16, 5.87, 6.15, 6.08, 6.14, 6.16]	[76.12, 76.21, 76.19, 76.28, 76.29, 76.08]	[89.27, 89.27, 89.04, 89.14, 89.23, 89.11]	[21.31, 21.46, 21.52, 21.22, 21.54, 21.6]	[4.57, 4.86, 4.81, 4.78, 4.63, 4.73]
		5-7pm	[2.45, 2.46, 2.37, 2.54, 2.35, 2.29]	[6.12, 6.14, 6.4, 6.03, 6.43, 6.16]	[76.65, 76.47, 76.53, 76.78, 75.74, 76.03]	[89.03, 89.22, 88.88, 89.12, 89.03, 89.21]	[20.9, 21.07, 21.1, 20.68, 21.91, 21.68]	[4.85, 4.64, 4.72, 4.85, 4.54, 4.63]
		9-11pm	[2.55, 2.79, 2.29, 2.5, 2.34, 2.48]	[6.25, 5.97, 5.83, 5.87, 5.85, 6.09]	[76.18, 75.99, 76.36, 76.67, 76.86, 76.49]	[88.75, 88.83, 89.03, 88.95, 88.87, 89.09]	[21.27, 21.22, 21.35, 20.83, 20.8, 21.03]	[5.0, 5.2, 5.14, 5.18, 5.28, 4.82]
	Primary	3-5am	[2.85, 2.68, 3.1, 3.14, 3.06, 3.13]	[6.06, 6.04, 5.61, 5.27, 5.45, 5.65]	[73.11, 73.29, 73.02, 73.45, 73.56, 73.64]	[87.88, 88.19, 88.22, 88.24, 87.93, 87.98]	[24.04, 24.03, 23.88, 23.41, 23.38, 23.23]	[6.06, 5.77, 6.17, 6.49, 6.62, 6.37]
		8-10am	[2.52, 2.16, 2.17, 2.36, 2.3, 2.34]	[6.34, 6.13, 6.54, 6.5, 6.79, 6.22]	[73.89, 74.93, 74.89, 74.26, 74.37, 73.94]	[88.51, 88.46, 88.57, 88.8, 88.85, 89.09]	[23.59, 22.91, 22.94, 23.38, 23.33, 23.72]	[5.15, 5.41, 4.89, 4.7, 4.36, 4.69]
		12-14am	[2.81, 2.4, 2.34, 2.47, 2.21, 2.21]	[6.4, 6.15, 6.38, 6.43, 6.42, 6.27]	[74.27, 74.49, 75.07, 74.67, 74.43, 74.92]	[88.98, 89.09, 88.67, 88.71, 88.96, 89.06]	[22.92, 23.11, 22.59, 22.86, 23.36, 22.87]	[4.62, 4.76, 4.95, 4.86, 4.62, 4.67]
		5-7pm	[2.59, 2.63, 2.4, 2.41, 2.18, 2.27]	[6.12, 6.16, 6.67, 6.01, 6.51, 6.21]	[74.2, 74.5, 74.73, 75.23, 73.88, 74.37]	[89.31, 89.2, 88.66, 89.37, 89.03, 89.19]	[23.21, 22.87, 22.87, 22.36, 23.94, 23.36]	[4.57, 4.64, 4.67, 4.62, 4.46, 4.6]
		9-11pm	[2.79, 2.87, 2.03, 2.42, 2.35, 2.43]	[6.69, 6.14, 6.02, 5.93, 5.92, 6.36]	[74.09, 74.34, 74.81, 75.03, 75.65, 74.78]	[88.21, 88.68, 88.88, 89.07, 88.59, 88.96]	[23.12, 22.79, 23.16, 22.55, 22.0, 22.79]	[5.1, 5.18, 5.1, 5.0, 5.49, 4.68]

Urban road network	Secondary	3-5am	[2.41, 2.86, 3.45, 3.02, 3.02, 2.94]	[5.88, 5.61, 5.51, 5.3, 5.39, 5.3]	[72.15, 71.54, 70.98, 70.71, 72.03, 71.66]	[87.66, 88.39, 87.51, 87.8, 87.27, 88.14]	[25.44, 25.6, 25.57, 26.27, 24.95, 25.4]	[6.48, 6.0, 6.98, 6.9, 7.34, 6.56]
		8-10am	[2.61, 2.09, 2.57, 2.11, 1.97, 2.37]	[6.25, 6.19, 6.32, 6.65, 5.88, 6.14]	[73.94, 73.96, 74.12, 74.18, 73.43, 72.94]	[88.12, 88.75, 88.51, 88.56, 89.46, 89.33]	[23.45, 23.95, 23.31, 23.71, 24.6, 24.69]	[5.63, 5.06, 5.17, 4.79, 4.68, 4.53]
		12-14am	[2.01, 2.33, 2.19, 2.41, 2.07, 2.13]	[6.3, 6.13, 6.07, 5.96, 6.02, 6.01]	[73.53, 73.79, 73.51, 73.9, 74.12, 73.15]	[89.31, 89.19, 89.55, 89.09, 89.28, 89.41]	[24.46, 23.88, 24.3, 23.69, 23.81, 24.72]	[4.39, 4.68, 4.38, 4.95, 4.7, 4.58]
		5-7pm	[2.29, 2.09, 2.47, 2.31, 2.53, 1.97]	[6.51, 6.51, 6.15, 6.19, 6.61, 6.74]	[74.16, 73.63, 73.92, 74.3, 73.57, 72.96]	[88.49, 88.87, 89.06, 88.93, 89.19, 88.62]	[23.55, 24.28, 23.61, 23.39, 23.9, 25.07]	[5.0, 4.62, 4.79, 4.88, 4.2, 4.64]
		9-11pm	[2.59, 2.7, 2.03, 2.29, 1.91, 2.39]	[6.16, 6.1, 5.61, 6.23, 5.74, 6.75]	[72.95, 73.04, 74.14, 73.63, 73.77, 73.57]	[88.82, 88.41, 89.51, 88.65, 89.12, 88.62]	[24.46, 24.26, 23.83, 24.08, 24.32, 24.04]	[5.02, 5.49, 4.88, 5.12, 5.14, 4.63]
	Tertiary	3-5am	[2.79, 2.97, 2.6, 3.16, 3.46, 3.22]	[6.19, 6.22, 5.85, 5.89, 5.54, 5.2]	[70.21, 70.68, 71.24, 70.05, 71.51, 71.67]	[87.15, 87.34, 87.52, 87.32, 87.52, 88.01]	[27.0, 26.35, 26.16, 26.79, 25.03, 25.11]	[6.66, 6.44, 6.63, 6.79, 6.94, 6.79]
		8-10am	[2.16, 2.32, 2.41, 2.06, 2.05, 1.95]	[6.56, 6.35, 6.87, 6.45, 6.34, 6.89]	[73.53, 74.54, 72.24, 75.0, 74.19, 73.75]	[87.94, 88.18, 87.47, 88.66, 88.78, 88.33]	[24.31, 23.14, 25.35, 22.94, 23.76, 24.3]	[5.5, 5.47, 5.66, 4.89, 4.88, 4.78]
		12-14am	[2.27, 2.38, 2.03, 2.06, 1.84, 2.06]	[6.49, 6.04, 6.71, 6.54, 6.45, 6.86]	[72.91, 73.4, 72.73, 72.88, 72.24, 72.75]	[88.63, 88.52, 88.29, 88.75, 88.68, 88.19]	[24.82, 24.22, 25.24, 25.06, 25.92, 25.19]	[4.88, 5.44, 5.0, 4.71, 4.87, 4.95]
		5-7pm	[2.33, 2.16, 2.13, 2.43, 1.94, 2.27]	[6.46, 6.57, 6.92, 6.77, 6.99, 6.54]	[74.15, 73.54, 73.24, 74.34, 72.11, 73.18]	[88.58, 88.74, 88.11, 88.04, 88.37, 88.7]	[23.52, 24.3, 24.63, 23.23, 25.95, 24.55]	[4.96, 4.69, 4.97, 5.19, 4.64, 4.76]
		9-11pm	[2.45, 2.35, 2.53, 2.14, 2.45, 2.16]	[6.46, 6.56, 6.26, 6.25, 6.28, 6.27]	[73.4, 73.38, 72.62, 74.11, 72.94, 73.34]	[88.44, 88.17, 88.2, 88.15, 88.4, 88.75]	[24.15, 24.27, 24.85, 23.75, 24.61, 24.5]	[5.1, 5.27, 5.54, 5.6, 5.32, 4.98]
		3-5am	[2.53, 2.45, 3.14, 2.76, 2.85, 3.1]	[5.56, 5.43, 5.51, 5.56, 5.22, 5.16]	[78.45, 78.45, 77.96, 77.38, 77.82, 76.78]	[88.35, 88.58, 88.65, 88.2, 88.25, 88.34]	[19.02, 19.1, 18.9, 19.86, 19.33, 20.12]	[6.09, 5.99, 5.84, 6.24, 6.53, 6.5]
		8-10am	[2.77, 3.12, 2.57, 2.58, 2.22, 2.7]	[6.1, 6.06, 6.42, 5.72, 6.22, 6.35]	[79.99, 77.97, 78.87, 80.06, 79.13, 79.42]	[88.31, 88.38, 87.98, 89.28, 88.55, 88.6]	[17.24, 18.91, 18.56, 17.36, 18.65, 17.88]	[5.59, 5.56, 5.6, 5.0, 5.23, 5.05]

	Residential	12-14am	[2.68, 2.57, 2.55, 2.74, 2.36, 2.58]	[5.84, 5.66, 6.2, 5.89, 6.39, 6.05]	[78.28, 77.86, 78.03, 78.68, 78.88, 78.62]	[89.35, 89.4, 88.53, 89.33, 88.96, 88.84]	[19.04, 19.57, 19.42, 18.58, 18.78, 18.8]	[4.81, 4.94, 5.27, 4.78, 4.65, 5.11]
		5-7pm	[2.62, 2.45, 2.47, 2.79, 3.08, 2.93]	[6.1, 6.29, 6.12, 6.11, 6.49, 5.8]	[78.83, 79.19, 79.04, 78.85, 77.88, 78.26]	[88.42, 89.01, 88.76, 88.58, 88.37, 89.01]	[18.55, 18.36, 18.49, 18.36, 19.04, 18.81]	[5.48, 4.7, 5.12, 5.31, 5.14, 5.19]
		9-11pm	[2.47, 3.06, 2.22, 3.12, 2.51, 3.12]	[5.93, 5.99, 5.68, 5.77, 5.98, 5.75]	[79.11, 78.68, 78.64, 79.23, 79.48, 78.62]	[88.92, 88.62, 88.94, 88.44, 88.56, 88.85]	[18.42, 18.26, 19.14, 17.65, 18.01, 18.26]	[5.15, 5.39, 5.38, 5.79, 5.46, 5.4]
Freeway network	ALL	8-10am	[4.39, 3.17, 3.54, 3.9, 3.54, 2.56]	[5.23, 4.76, 5.37, 4.42, 4.28, 4.01]	[69.02, 70.12, 68.78, 69.15, 69.63, 70.85]	[86.96, 87.7, 87.43, 87.5, 89.88, 89.95]	[26.59, 26.71, 27.68, 26.95, 26.83, 26.59]	[7.81, 7.54, 7.2, 8.08, 5.84, 6.04]
		12-14am	[2.07, 2.2, 2.93, 3.17, 2.56, 2.68]	[4.55, 4.01, 4.21, 5.37, 4.42, 4.08]	[70.86, 68.78, 69.27, 69.02, 70.0, 70.12]	[88.32, 88.86, 90.42, 88.11, 89.2, 87.77]	[27.07, 29.02, 27.8, 27.81, 27.44, 27.2]	[7.13, 7.13, 5.37, 6.52, 6.38, 8.15]
		5-7pm	[3.9, 4.51, 4.39, 4.63, 5.12, 4.88]	[4.82, 4.42, 5.84, 4.82, 4.35, 4.62]	[70.85, 70.61, 69.27, 70.24, 68.41, 67.2]	[88.45, 88.58, 87.16, 89.4, 87.84, 88.65]	[25.25, 24.88, 26.34, 25.13, 26.47, 27.92]	[6.73, 7.0, 7.0, 5.78, 7.81, 6.73]

Table S6. Proportions of Spatial-temporal correlations of travel speeds.

Road network type	Road type	Time ranges	Significant negative(%)	Insignificant(%)	Significant positive(%)
Urban road network	All	3-5am	1.13	56.93	41.94
		8-10am	0.5	62.94	36.56
		12-14am	0.72	72.02	27.26
		5-7pm	0.58	67.32	32.1
		9-11pm	0.7	71.71	27.59
	Primary	3-5am	1.12	58.91	39.97
		8-10am	0.56	61.12	38.32
		12-14am	0.79	72.3	26.91
		5-7pm	0.7	65.72	33.58
		9-11pm	0.83	72.07	27.1
	Secondary	3-5am	0.98	56.06	42.96
		8-10am	0.49	63.54	35.97
		12-14am	0.76	72.63	26.61
		5-7pm	0.61	67.59	31.8
		9-11pm	0.7	71.81	27.49
	Tertiary	3-5am	1.27	53.4	45.33
		8-10am	0.54	64.4	35.06
		12-14am	0.75	70.24	29.01
		5-7pm	0.54	67.74	31.72
		9-11pm	0.71	69.65	29.64
	Residential	3-5am	1.29	53.22	45.49
		8-10am	0.38	61.57	38.04
		12-14am	0.57	69.29	30.14
		5-7pm	0.47	66.34	33.19
		9-11pm	0.51	70.11	29.38
Freeway network	ALL	8-10am	1.65	45.55	52.8
		12-14am	0.62	41.91	57.47
		5-7pm	2.06	44.04	53.9

Table S7. Proportions of Spatial-temporal partial correlations of travel speeds.

Road network type	Road type	Time ranges	Significant negative(%)	Insignificant(%)	Significant positive(%)
Urban road network	All	3-5am	1.83	67.83	30.34
		8-10am	0.85	75.27	23.88
		12-14pm	1.06	77.86	21.08
		17-19pm	0.91	76.16	22.93
		21-23pm	1.07	77.85	21.08
	Primary	3-5am	1.81	68.42	29.77
		8-10am	0.98	73.42	25.6
		12-14pm	1.17	77.9	20.93
		17-19pm	1.06	74.63	24.31
		21-23pm	1.23	77.97	20.8
	Secondary	3-5am	1.81	68.15	30.04
		8-10am	0.9	76.4	22.7
		12-14pm	1.13	78.65	20.22
		17-19pm	0.94	77.07	21.99
		21-23pm	1.09	78.56	20.35
	Tertiary	3-5am	1.97	65.53	32.5
		8-10am	0.86	75.8	23.34
		12-14pm	1.08	76.4	22.52
		17-19pm	0.85	76.23	22.92
		21-23pm	1.1	76.25	22.65
	Residential	3-5am	2.07	65.96	31.97
		8-10am	0.68	74.37	24.95
		12-14pm	0.88	75.75	23.37
		17-19pm	0.79	75.23	23.98
		21-23pm	0.82	76.1	23.08
Freeway network	ALL	8-10am	2.61	61.76	35.63
		12-14pm	1.87	61.66	36.47
		17-19pm	3.36	60	36.64

Table S8. Differences in proportions of different types of correlations between different types of road links. Given a correlation type (temporal, temporal partial, etc) and a road link (primary, secondary, tertiary, and residential), we obtain its proportions of significant positive, insignificant, and significant negative correlations in different time ranges and time periods. This table presents the mean and the standard deviation of differences of these proportions between any two types of road links. For example, the 0.08 (0.11) marked in yellow represents that, for the temporal correlation, the mean (standard deviation) of differences of the proportions of significant negative correlations between primary and secondary links is 0.08% (0.11%).

**Correlation type**	**Two types of road links for comparison**	**Significant negative (%)**		**Insignificant (%)**		**Significant positive (%)**	
		**mean**	**std**	**mean**	**std**	**mean**	**std**
Temporal	primary-secondary	0.08	0.11	3.73	2.63	3.75	2.67
	primary-tertiary	0.22	0.29	6.55	4.07	6.57	4.28
	primary-residential	0.15	0.20	7.02	4.23	6.99	4.36
	secondary-tertiary	0.15	0.19	3.37	2.06	3.39	2.12
	secondary-residential	0.10	0.09	4.18	2.29	4.19	2.33
	tertiary-residential	0.14	0.11	1.01	0.62	1.02	0.66
Temporal partial	primary-secondary	0.90	1.49	1.05	0.98	0.71	0.69
	primary-tertiary	1.91	3.01	2.43	1.93	1.87	1.42
	primary-residential	1.65	2.86	2.89	2.01	2.41	1.93
	secondary-tertiary	1.06	1.57	1.42	1.24	1.17	1.03
	secondary-residential	0.93	1.41	2.02	1.53	1.72	1.66
	tertiary-residential	0.31	0.32	0.86	0.68	0.55	0.69
Spatial (directly adjacent cases)	primary-secondary	0.17	0.12	1.45	1.15	1.44	1.21
	primary-tertiary	0.16	0.13	2.51	1.22	2.48	1.14
	primary-residential	0.21	0.13	2.77	1.44	2.82	1.40
	secondary-tertiary	0.20	0.17	1.46	1.04	1.42	1.02
	secondary-residential	0.21	0.13	2.42	1.19	2.40	1.28
	tertiary-residential	0.14	0.10	1.75	1.13	1.83	1.17
Spatial (indirectly adjacent cases)	primary-secondary	0.18	0.12	1.02	0.75	1.02	0.79
	primary-tertiary	0.14	0.10	1.82	1.32	1.78	1.37
	primary-residential	0.26	0.14	1.98	1.22	1.77	1.25
	secondary-tertiary	0.15	0.13	1.04	0.81	1.02	0.87
	secondary-residential	0.21	0.16	1.18	0.76	1.04	0.71
	tertiary-residential	0.20	0.13	0.73	0.75	0.70	0.80
Spatial partial (directly	primary-secondary	0.21	0.18	1.09	0.63	1.21	0.66
	primary-tertiary	0.27	0.16	1.51	0.88	1.64	0.83
	primary-residential	0.31	0.24	4.29	0.77	4.47	0.73

adjacent cases)	secondary-tertiary	0.27	0.19	0.70	0.54	0.71	0.59
	secondary-residential	0.38	0.25	5.37	0.81	5.67	0.69
	tertiary-residential	0.48	0.27	5.75	1.00	6.08	0.86
Spatial partial (indirectly adjacent cases)	primary-secondary	0.27	0.20	0.40	0.20	0.26	0.20
	primary-tertiary	0.30	0.19	0.53	0.32	0.32	0.21
	primary-residential	0.32	0.23	0.34	0.25	0.35	0.27
	secondary-tertiary	0.39	0.23	0.57	0.39	0.30	0.18
	secondary-residential	0.28	0.26	0.45	0.30	0.43	0.29
	tertiary-residential	0.48	0.21	0.51	0.32	0.27	0.20
Spatial-temporal	primary-secondary	0.09	0.04	1.55	1.07	1.56	1.07
	primary-tertiary	0.10	0.06	3.06	1.31	3.02	1.26
	primary-residential	0.22	0.05	2.35	1.92	2.34	1.95
	secondary-tertiary	0.09	0.10	1.64	0.97	1.58	0.93
	secondary-residential	0.19	0.07	2.22	0.76	2.28	0.72
	tertiary-residential	0.13	0.07	1.16	0.93	1.20	1.02
Spatial-temporal partial	primary-secondary	0.08	0.05	1.41	1.09	1.33	1.07
	primary-tertiary	0.14	0.04	2.02	0.53	1.96	0.48
	primary-residential	0.31	0.05	1.61	0.71	1.58	0.90
	secondary-tertiary	0.07	0.05	1.72	0.83	1.73	0.78
	secondary-residential	0.23	0.04	2.28	0.37	2.41	0.47
	tertiary-residential	0.16	0.08	0.73	0.45	0.90	0.42
